# Detection and control of *Ganoderma boninense*: strategies and perspectives

**DOI:** 10.1186/2193-1801-2-555

**Published:** 2013-10-24

**Authors:** Roozbeh Hushiarian, Nor Azah Yusof, Sabo Wada Dutse

**Affiliations:** Institute of Bioscience, Universiti Putra Malaysia, Serdang, Selangor 43400 UPM Malaysia; Department of Chemistry, Faculty of Science, Universiti Putra Malaysia, Serdang, Selangor 43400 UPM Malaysia; Institute of Advanced technology, Universiti Putra Malaysia, Serdang, Selangor 43400 UPM Malaysia; Department of Science Laboratory Technology, Hussaini Adamu Federal Polytechnic, Kazaure, Nigeria

**Keywords:** *Ganoderma boninense*, Oil palm tree, Basal stem rot disease

## Abstract

The oil palm, an economically important tree, has been one of the world’s major sources of edible oil and a significant precursor of biodiesel fuel. Unfortunately, it now faces the threat of a devastating disease. Many researchers have identified *Ganoderma boninense* as the major pathogen that affects the oil palm tree and eventually kills it. But identification of the pathogen is just the first step. No single method has yet been able to halt the continuing spread of the disease. This paper discusses the modes of infection and transmission of *Ganoderma boninense* and suggests techniques for its early detection. Additionally, the paper proposes some possible ways of controlling the disease. Such measures, if implemented, could contribute significantly to the sustainability of the palm oil industry in South East Asia.

**Electronic supplementary material:**

The online version of this article (doi:10.1186/2193-1801-2-555) contains supplementary material, which is available to authorized users.

## Introduction

Fungi which rot and eventually kill oil palm trees may be costing some South East Asian countries US$500 million a year. Indonesia and Malaysia jointly produce 84% of the world’s total palm oil (Khairil and Hasmadi 2010; Ommelna et al. 2012). It has been reported that the economic loss caused by this pathogen is between RM225 million to RM1.5 billion (up to 500 million USD) a year (Ommelna et al. 2012; Arif et al. 2011).

The oil palm tree (*Elaeis guineensis* jacq.) originates from West Africa but was introduced to South East Asia by the British in the early 1870s. Oil palm trees start bearing fruit after 30 months of planting and are productive for 20 to 30 years. Needing just 0.26 hectares of land to produce one tone of oil, they are the most efficient oil-bearing crop in the world (Idris 2013). Unfortunately, infection with fungi has caused a decline in the productivity of oil palms and subsequently the palm oil industry, and created significant concern (Hartley 1967; Turner 1981).

A wide variety of fungi can affect and decay all types of woody plants and trees by colonizing in wounds on trunks, branches and roots. The disease developed is commonly called *wood rot disease* (Blanchette 1984) or *heartwood* when it is limited to the centre of older trees. Although the process may be very slow, it is estimated that the damage caused to timber trees by fungi, particularly in forests, is more than all other natural disasters combined, including insects and fire (Lonsdale et al. 2008).

Wood rot disease is classified according to each of the three areas affected - root rots, root and butt rots and stem rots (Arya and Perelló 2010). All wood rots grow inside the wood cells and degrade cell wall components. *Brown rot fungi* use cell wall polysaccharides and leave the lignin, while *white rot fungi* degrade lignin as well as all other wood components (Adaskaveg et al. 1993; Adaskaveg et al. 1991).

Although other fungi belonging to the Ascomycetes family can also decay wood, the species of fungi mostly responsible for developing complex wood rot diseases are Basidiomycetes and particularly the Ganoderma family (Khairuddin 1990; Rao 1990). Several different species of Ganoderma such as *Heterobasidion*, *Polyporus*, *Inonotus*, *Laetiporus*, *Phellinus*, *Chondrostereum*, *Peniophora*, *Lenzites*, *Pleurotus*, *Schizophyllum*, and *Trametes* are are responsible for a wide range of wood rots in a variety of trees (Seo and Kirk 2000; Moncalvo 2000). In all, at least seventy-five different examples of Ganoderma have been collected from twenty-one separate locations in Malaysia alone (Turner 1981). However, it is *Ganoderma boninense* which has been identified as the major disease of oil-palm trees (Khairuddin 1990; Rao 1990).

*Ganoderma boninense* (*G. boninsense*) causes both basal stem rot (BSR)and upper stem rot (USR) and remains South East Asia’s most devastating oil palm diseases with direct loss of the stand, reduced yield of diseased palms and the resultant requirement for earlier replanting (Flood et al. 2002). Once young palms show symptoms of the disease they usually die within 1 or 2 years, while mature trees can survive for only another 3 or so years (Corley and Tinker 2003).

Although it has been clearly identified as the main cause of the disease in oil palms, strategies for the early detection and control of *G. boninense* are still immature. The purpose of this paper is to outline the existing strategies, to evaluate their effectiveness and to suggest ways in which the spread of this destructive fungus might best be addressed.

### Infection and transmission

Considerable controversy remains but identifying the route of infection and the extent of pathogen diversity is critical to the development of effective disease control and plantation management (Cooper et al. 2011).

Ganoderma is characterized by basidiocarps large, perennial, woody brackets which are lignicolous and leathery, sometimes with a stem. The fruit bodies typically grow in a fan- or hoof-like form on the trunks of trees. They have double-walled, truncated spores with yellow to brown ornamented inner layers.

Delays in detection have been compounded by confusion between *G. zonatum,* weakly pathogenic to oil palm, and *G. boninense* the major pathogen (Pilotti 2005). High intraspecific variability found among *G. boninense* isolated from either neighbouring or distant trees supports the belief that sexual reproduction plays an important role in the epidemiology of Basal Stem Rot disease (Pilotti et al. 2003). Although there have been differences found among isolates from the same tree pointing to different strains (Miller et al. 1999), some researchers believe that the disease is a single taxon (Bridge et al. 2000).

Another reason the disease has not been well-detected in its early stages is because its cycle consists of a number of alternative and consecutive events: Firstly, there must be an injury to expose the wood. The cells around the injured area oxidize and discolour due to biochemical changes. This discolouration can develop further if the wound remains open to the many microorganisms which can land and grow on the moisture. The next most likely step is for bacteria and Ascomycetes or imperfect fungi to live on the wound, resulting in further discolouration, wetness of the area and erosion of parts of the cell wall. This is referred to as *wetwood, redheart*, or *blackheart*. Finally, as the wood-rotting fungi (Basidiomycetes) integrate, digestion of cell wall components begins. These microorganisms, however, are confined to the area which is already undergone the biochemical processes followed by bacterial reaction, Ascomycetes and imperfects. While these remain in tissues inside the endodermis in the initial stages their hyphae may be detected in different cells and tissues in advanced stages of disease. Infection of the stem leads to the formation of 'black lines’ within the infected tissues (Ariffin et al. 1989,1991).

Ariffin and his colleagues had earlier revealed that the black line observed in the stem of oil palm infected with *G. boninense* is caused by a single mycelium and thus emphasizes the fungal origin of its formation (Ariffin et al. 1989). As presence of fungal hyphae almost exclusively on one side of the black line precludes the possibility of a dual infection involving another fungus and clearly indicates that *G. boninense* is the sole fungus present. Similarly, based on *in vitro*, morphological studies, *G. boninense* has been associated with the rotten upper stem. Flood and her colleagues stated that USR invariably causes discrete lesions originating from the frond base and spreading in successive wave of rot, each delimited by the brown or black line (Flood et al. 2002).

The ratio of USR to BSR in plantation estates ranges from 1:10 to 1:1 with some having an incidence of USR exceeding that of BSR (Hasan et al. 2005). In some commercial fields in Indonesia, the incidence of USR is higher than incidence of BSR (Flood et al. 2002) but its incidence in most Malaysian plantations is less than 5% (Turner and Gillbanks 1974).

Upper stem rot has symptoms similar to those of spear rot, bud rot, bunch rot and basal stem rot caused by root diseases. Lower leaves first become yellow and die from the tip to the base. This condition progresses to the middle of the crown, finally affecting the spear leaves. The stem tissues show a brown rot even when the roots of the palm are not affected (Hasan et al. 2005).

The symptoms of Basal stem rot are decay of the bottom of the stem from where basidiocarps emerge and sometimes also decay of the roots. Stem rotting restricts the uptake of water and nutrients to the fronds, causing chlorosis. When the disease is more advanced, the older fronds wilt and hang down to form a skirt around the trunk (Turner and Gillbanks 1974). Other observable symptoms are flattening of the crown and spear leaves that have not opened. In the most severe cases the stem might even fracture (Rees et al. 2012).

The whole process of discolouration and decay occurs within a few years to a few decades and is more common in older trees. Infection has been reported in four or five year old trees in replanted areas (Singh 1990) as well as in areas under plantation with coconut where *G. boninense* was found attacking seedlings and palms less than a year old in the nursery (Susanto 2009). The process does not necessarily complete and may stop at any stage due to quick healing of the wound, natural resistance of the wood or some antagonistic relationship between microorganisms. However, older trees are more likely to get injured during their lifespan and may be exposed to the disease process repeatedly.

Ganoderma spreads in the soil through roots and through the air. Compatibility studies have shown that fungi collected from the same field or area may have different origins so mycelial growth is probably not the only method of transmission of disease among the trees (Miller 1995). Although the development and spread of wood rots may differ from fungus to fungus and according to the type of tree, there are similarities. Basidiomycetes, like Ganoderma, have two strategies for reproduction: spores and mycelia. Other root-infecting basidiomycetes such as *Heterobasidi onannosum* spread from tree to tree through the soil by vegetative growth, often as one genet (Woodward et al. 1998) but studies of *G. boninense* have found that there is such diversity in oil palm plantations that infections by more than one genotype through sexual recombination with dispersal by spread of basidiospores has arisen (Pilotti et al. 2002).

Although they are certainly involved and there is evidence that they are able to germinate on cuts stems in plantations, there have been no successful infections of oil palms with basidiospores (Hasan et al. 2005; Idris 2013; Cooper et al. 2011). The reasons for this would seem to be *G. boninsense’* low aggressiveness and the need for large inocula (Rees et al. 2007).

Wind, rain and insects all assist to carry spores to wounds on trees, most commonly those which have been cut. In particular, the *Oryctes* beetle (Turner and Incorporated Society of Planters. 1981) and larvae of the *Sufetula* spp caterpillar play at least a small role in the spreading of Ganoderma spores (Genty et al. 1976). Experiments in which an enormous number of Ganoderma spores were released in a field but did not infect most trees (Ho and Nawawi 1986) have indicated that infected tissues in the soil are more likely to spread the disease to healthy roots than airborne spores.

Thus, although not all scientists agree on how oil palms are infected and how the disease spreads, and indeed many studies including attempts to inoculate and infect oil palms with basidiospores have to date not been successful (Hasan and Flood 2003), there is, nevertheless, widespread acceptance that *G. boninsense* is the cause of both BSR and the less common USR (Navaratnam and Chee 1965; Lim et al. 1992; Sariah et al. 1994; Hasan and Turner 1998; Lim and Fong 2005; Breton et al. 2006).

### Detection

Observation of such symptoms in the field as mature leaves wilting and falling through malnutrition or the presence of basidiomata of the pathogen on the tree was the only method of diagnosing disease in the early days (Lelong et al. 2010). The other early diagnostic method was drilling into diseased material in the tree for sampling and then using either a colorimetric method using ethlylenediaminetetraacetic acid (EDTA) (Natarajan et al. 1986) or a semi-selective media to cultivate Ganoderma on agar plates (Darus et al. 1993). Both of these methods were time-consuming and not very accurate and it is now recognized that by the time basidiomata is apparent the disease is already well-established. Indeed there were no accurate techniques for detecting subclinical infections prior to the beginning of this century (Utomo and Niepold 2000).

The earliest molecular attempts were based on immunoassay. Antibodies were employed to detect Ganoderma in culture media (Reddy and Ananthanarayanan 1984; Darmono et al. 1993; Darmono and Suharyanto 1995). But many issues stood in the way of accurate and efficient techniques, including the lack of proper taxonomy information and confusion between some species of the genus (Moncalvo 2000; Ryvarden 1995; Paterson 2006a).

In an enzyme-linked immunosorbent assay, (ELISA), polyclonal antibodies were employed and showed relatively good negative results. Unfortunately, the test proved to be not species-specific as antibodies cross-reacted with other species (Utomo and Niepold 2000).

A study in which crude mycelium extract of *G. boninense* was used as an immunogene to generate monoclonal antibodies was found to give significantly better results than polyclonal antisera (Shamala et al. 2006) but further work is needed to produce monoclonal antibodies more specific to pathogenic species.

A promising Ganoderma-selective medium has facilitated sampling from the field for further studies (Ariffin et al. 2000). A more accurate alternative molecular diagnosis technique is to target oligonucleotides particularly DNA.

To design a reliable diagnostic molecular technique based on DNA sequencing, it is essential to have sufficient sequencing data. Table 1 shows that apart from manganese-superoxide dismutase, an enzyme related to the antioxidant defence mechanism of the cell, other available sequences are either related to ribosomal RNA (rRNA) or are designed molecular techniques for detection of the microorganism.

**Table 1:** **Available nucleotide sequences of** ***Ganoderma boninense*** **in databases**

Accession number	Sequence length (bp)	Product	Source
AAB16771	426	Manganese-superoxide dismutase	Strain RSH RS
U56128	683	Manganese-superoxide dismutase	Strain RSH RS
JQ665226	745	18S rDNA	Isolate G1
JQ665227	743	18S rDNA	Isolate G2
JQ665228	744	18S rDNA	Isolate G3
JQ665229	745	18S rDNA	Isolate G4
JQ665230	735	18S rDNA	Isolate G5
JQ665231	743	18S rDNA	Isolate G6
JQ665232	740	18S rDNA	Isolate G7
JQ665233	745	18S rDNA	Isolate G8
JQ665234	742	18S rDNA	Isolate G9
JQ665235	740	18S rDNA	Isolate G10
JQ665236	737	18S rDNA	Isolate G11
JQ665237	741	18S rDNA	Isolate G12
JQ665238	740	18S rDNA	Isolate G14
AF255198	1729	18S rDNA	Strain FA-PP28
FJ154775	884	18S rDNA	Isolate GR376
EU701010	297	18S rDNA, misc_RNA (ITS 1)	Strain FA5035
EU841913	297	18S rDNA, misc_RNA (ITS 1)	Strain FA5017
X78749	238	RS rDNA and ITS 1	Strain RS
X78770	207	RS rDNA and ITS 2	Strain RS
X78777	1458	25S rDNA	Strain RS
E38237	56	Method for detection	-
E38238	20	Method for detection	-
E38239	20	Method for detection	-
E38240	18	Method for detection	-
BD082757	616	Method for detection	-
BD082758	616	Method for detection	-
BD082759	612	Method for detection	-
BD082761	24	Method for detection	Synthetic construct
BD082762	24	Method for detection	Synthetic construct
BD082763	24	Method for detection	Synthetic construct
BD082764	23	Method for detection	Synthetic construct
BD082765	20	Method for detection	Synthetic construct
BD082766	22	Method for detection	Synthetic construct

The small ribosomal subunit RNA (in prokaryotes 16S and in eukaryotes 18S) gene is one of the most important molecular markers with a range of applications in biodiversity screening, phylogenetic analyses and evolutionary studies (Meyer et al. 2010). It is known to be a high similarity among sequences of rRNA (Kwon et al. 1991), particularly the 18S subunit and its secondary structure, within different species, and thus may be a reliable target for classification purposes (Neefs et al. 1990). A phylogenetic study of Ganoderma using single locus mt SSU rDNA led to dividing Ganoderma into six distinct monophyletic groups and indicated that complex situations related to the geographical region and the pathogen-host relationship must be considered as well as the phylogenetic relationships (Soon Gyu and Jung 2004). Nuclear 18S rDNA analysis may also be used to provide molecular evidence on the long distance dispersal of Ganoderma across the southern hemisphere (Moncalvo and Buchanan 2008) and has already shown the diversity of wood-decaying fungi in India (Singh et al. 2013).

**Figure 1:**
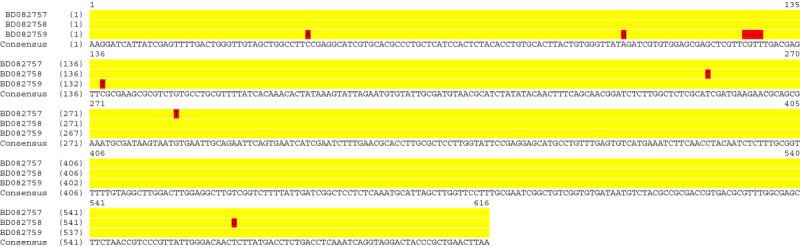
**Multiple sequence alignment of part of 18S rDNA of** ***G. boninense*** **described in a patented method for detection.**

A review on available data in the genebank following DNA sequences with accession numbers: BD082757, BD082758 and BD082759 which are retrieved from a patented method for detection (Sakamoto et al. 2001) are aligned and shown to have little difference (Figure 1).

**Figure 2:**
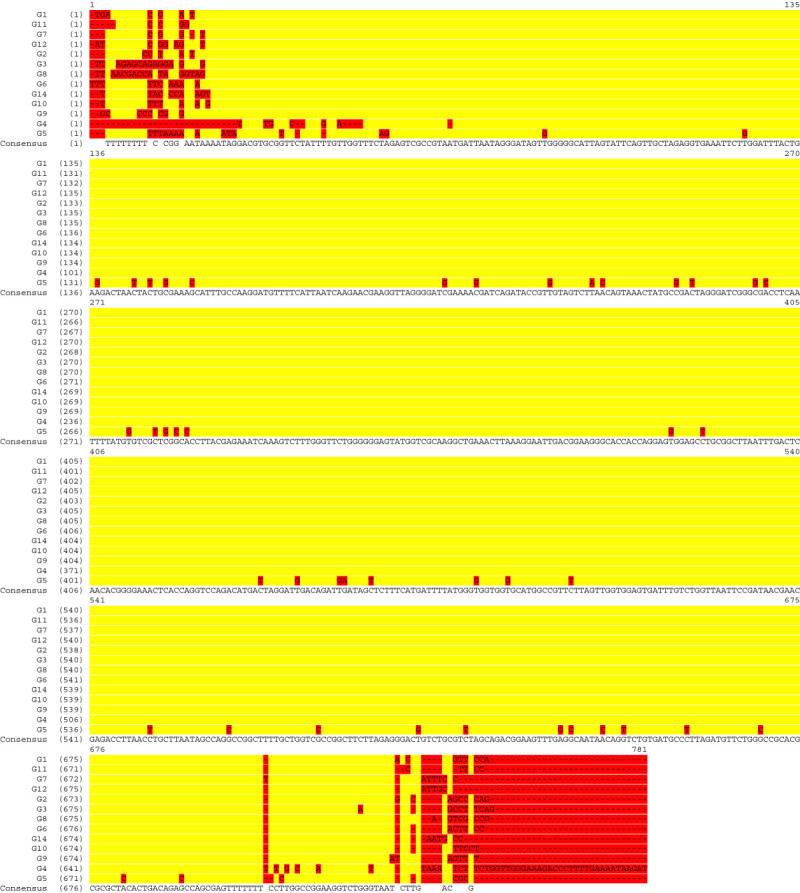
**Multiple sequence alignment of partial 18S rDNA from isolates G1, G2, G3, G4, G5, G6, G7, G8, G9, G10, G11, G12, G14.**

A multiple sequence alignment of fragments of 18S rDNA from different isolates in Malaysia (Kok et al. 2013) shows close similarity in the region between bases 22 and 708 (Figure 2). Isolate G5 with almost 7% difference can be differentiated from the others.

**Figure 3:**
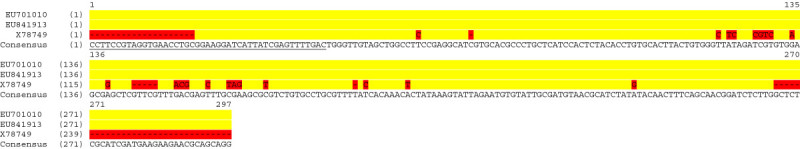
**Sequence alignment between partial ITS1segments with accession numbers EU701010, EU841913 and X78749.**

Sequences with accession numbers of EU701010 and EU841913 are 100% identical (Figure 3). They start with 45 bp of 18S rDNA.

**Figure 4:**
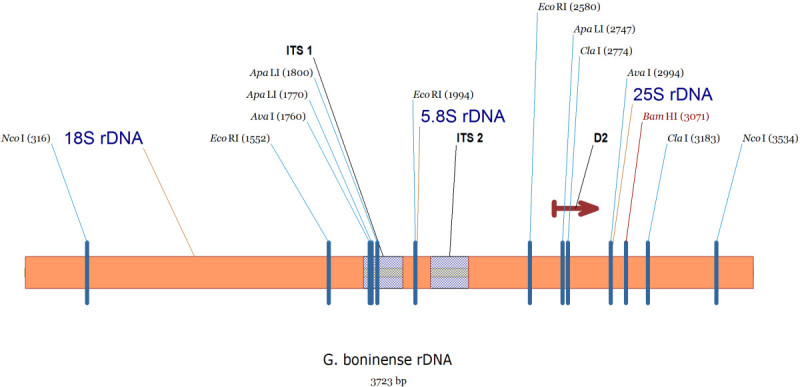
**Schematic map of nuclear rDNA with related restriction endonuclease sites.**

The alignment between the consensus sequences from Figures 1, 2 and 3 and the longest available sequence of 18S rRNA gene (1729 bp) with accession number AF255198 (Moncalvo and Buchanan 2008) combined with other sequences in Table 1 produced a map of rDNA which is illustrated in Figure 4. This map is completed by comparison of these data with Moncalvo and Buchanan’s findings and nearest relatives of *G. boninense*; *G. australe* and *G. pfeifferi* (Moncalvo et al. 1995). The D2 region in 25S rDNA is shown by the arrow. This region has been used for phylogenetic study and evolutionary relation between taxa and it has been shown that *G. boninense* and *G. pfeifferi* have just one nucleotide difference despite their 14 nucleotide difference in the Internal Transcribed Spacer (ITS) 1.

This information can be employed for the development of molecular techniques for detection purposes.

Polymerase Chain Reaction (PCR), by providing an opportunity for the amplification of certain regions of a template molecule (DNA or RNA), has been of great benefit to development and innovation in a variety of PCR-based techniques with diverse applications. PCR test using universal fungal primers for ITS region showed relatively more accuracy than ELISA (Utomo and Niepold 2000).

Even now, it is still worthwhile for oil plantation farmers to use a combination of both procedures – the ELISA test for bulk screening and, where the results are positive, a subsequent PCR test to verify the results and to isolate Ganoderma (Utomo and Niepold 2000).

One of the most practical PCR-based techniques in characterization and detection of pathogenic fungi in plants is Random Amplification of Polymorphic DNA (RAPD). Here several arbitrary short primers are used with a long genomic DNA template in the expectation that some of them will amplify and provide a profile of the template (Williams et al. 1990; Williams et al. 1993). RAPD, together with microsatellite analysis, provides a further technique known as Random Amplification Microsatellite (RAMS) (Hantula et al. 1996). RAPD and RAMS can be adjusted (Zakaria et al. 2005) and employed to analyze different isolates of *Ganoderma* spp. (Zakaria et al. 2009).

Applying the Restriction Fragment Length Polymorphism (RFLP) technique in both highly conserved and variable sequences of ITS of rDNA at the species level provides an opportunity to facilitate genetic variation studies (Nusaibah et al. 2011). Since there is more divergence observed in ITS 1 rather than ITS 2 (Moncalvo and Buchanan 2008), it is recommended designing an experiment based on ITS 1 sequences. Furthermore, unlike ITS 2 there are 3 restriction sites in ITS 1 region. A PCR-RFLP method based on some sequential differences between pathogenic and non-pathogenic *Ganoderma* spp. has been designed and reported to be both more accurate and less sensitive to contamination than RAPD and Amplified fragment length polymorphism (AFLP) (Utomo et al. 2005) although the importance of internal amplification controls (IAC) is ignored in this method (Paterson 2007). Using IAC is highly recommended to avoid false negative results in PCR methods (Hoorfar et al. 2004).

The Malaysian Palm Oil Board (MPOB) has worked extensively on the early detection of Ganoderma. After an attempt to design a PCR technique (Idris et al. 2003), the Board worked on a polyclonal antibody for ELISA technique (Idris and Rafidah 2008) and finally provided a multiplex PCR-DNA kit (Idris et al. 2010). However, the PCR technique is vulnerable to contamination and since some secondary metabolites can inhibit PCR (Paterson 2004; Mizushina et al. 1999), purification of the DNA prior to the reaction is crucial. Regardless, the alkaline DNA extraction is simpler and quicker than other DNA extraction methods combined with purification procedures (Utomo and Niepold 2000).

FISH and DNA microarray are interesting options to the DNA-based molecular techniques outlined above. An electrochemical DNA biosensor has been designed and adjusted for detection of *G. boninense* (Dutse et al. 2012; Dutse et al. 2013) but as for all direct molecular techniques, the preparation of representative samples and extraction of DNA remains a challenge.

Meanwhile, as long as molecular methods remain complex and time-consuming, other techniques like E-nose, tomography and Hyperspectral reflectral data continue to be explored. A recent study successfully investigated the biomarkers (volatile compounds) of diseased oil palms for the development of specific Molecularly Imprinted Polymer (MIP) sensors (Abdullah et al. 2012). This built on earlier studies which had used a commercially available electronic nose and an artificial neural network with 32 sensors for different trees. The odour profile recorded provided differentiating information that seems to be practical particularly if it is accompanied with chromatography techniques (Markom et al. 2009; Abdullah et al. 2012).

In another recent study, ergosterol, a membrane component of fungi, was investigated using high-performance liquid chromatography (HPLC). It is believed that quantification of this component accompanied by other techniques could provide a complex diagnosis system approaching the early detection aim (Mohd As’wad et al. 2011).

Another method to non-molecular approaches is a 2007 study undertaken by MohdShu’ud and his colleagues, who attempted to identify Ganoderma in oil plant stems from tomography images (Shu’ud et al. 2007). Expert rules were established in a Fuzzy Inference System and images from the trees triaged into 3 classifications: intact tissue, Ganoderma infection and infection other than Ganoderma. This model is obviously limited by the experts’ knowledge of infection patterns in oil palms and to make it more feasible for widespread use, further testing to improve the rules is required.

Yet another non-molecular diagnostic method, claimed by its authors to be less complex and expensive and indisputably less damaging to the tree itself, is the use of statistical modeling to classify canopy spectra into Ganoderma attack severity levels (Lelong et al. 2010). First, field protocols and measurements are taken, a combination of analyses is applied and finally an algorithm is developed which has already been able to assess 4 levels of disease severity with 94% accuracy. Although this tool seems promising, especially in identifying seriously diseased trees from healthy ones, further work needs to be undertaken to more accurately discern the intervening levels of infection in the trees.

Overall, it has been and continues to be very difficult to find a species-specific detection method.

### Control

The lack of sufficient data about *Ganoderma* spp. required for developing a reliable early stage diagnosis system inevitably leads to difficulty in control of the disease (Rolph et al. 2000). Currently, although it is impossible to manage a field free of pathogen (Sanderson et al. 2000), considerable reduction of the disease can be achieved through a proper management system of healthy stands to reduce the introduction of the pathogen to them. This is done by minimizing wounds on trees, by improving treatment and harvesting operations, and by clearing the old trees before extreme age susceptibility (Flood et al. 2000). It has been a routine practice to treat large wounds in other trees with paint or dressing and this seems just as applicable for oil palm trees, although its effectiveness in the prevention of decay is questionable. The use of fungicides tends to be another legitimate treatment for living stands, although this approach has yet to be studied and developed (Ariffin et al. 2000).

In fact, there are at least ten different techniques which have been used in attempts to manage the disease, with varying levels of success. Relatively ineffective measures include soil mounding, surgery and isolation trenching.

### Soil mounding

This method in which soil is heaped around the trunk to a height of 75 cm may prolongs the life of the tree but is not effective in controlling BSR (Lim et al. 1993; ChengTuck and Hashim 1997).

### Surgery

Cutting out the dead tissue/basidiocarps by hand with a chisel (Turner 1981) or with a mechanical back-hoe (Singh 1990) has been tried with generally mediocre results, with the exception of some moderate success in small plantations (Cooper et al. 2011; Marshall et al. 2004).

More successful methods include isolation trenching, sanitation and leaving soil fallow after ploughing.

Isolating a diseased tree or stump with a 2×2 meter trench has been found to be a useful method of delaying BSR by up to 14 years because it stops contacts between roots (Hasan and Turner 1998; Chung 2011).

### Sanitation or removal of diseased material

The removal of diseased materials is usually carried out in two situations – in existing plantings where BSR is evident, and at the replanting stage. For many years, burning the materials has been common but this is creating environmental issues in the region. Quarantining, shredding and isolating are other ways in which the roots of healthy palms can be prevented from coming into contact with those of diseased palms, stumps or roots (Singh 1990; Turner 1981; Khairuddin 1990; Idris et al. 2004; Chung 2011).

### Ploughing and harrowing

Large plantations often use 2 rounds of ploughing to 60cm depth and one round of harrowing to chop up the remaining roots in disease prone areas before planting new seedlings (Flood et al. 2000).

### Fallowing

Situations in which the soil has been left fallow in replanting systems have shown significant effect on later disease incidence. Further studies are continuing to assess the optimum length of fallow and the potential planting of other crops to balance the economic effect (Virdiana et al. 2010).

### Planting legume cover crops (LCC)

Mandatory in some plantations, the practice of planting ground covers, while controlling weeds and erosion, may in fact be introducing legume species which are themselves susceptible to *G. boninense* (Chung 2011) so must be undertaken with caution.

### Chemical treatments

Although investigated as an option, injection of fungicides such as hexaconazole is rarely practiced because this method has not shown to provide effective control (Chung 2011).

### Fertiliser

It is still too soon to ascertain how effective fertilisers are against BSR. Experimentation with macro- and micro-elements such as K, N and P have resulted in some positive change to disease levels and plant productivity (Singh 1990; Chung 2011).

### Biological control

Antagonistic fungi have been under investigation for a few decades after successful protection of stumps against *Fomesannosus* (Rishbeth 1959,1963) followed by commercial control of the disease. From early studies on the biological control of wood rotting fungi, species of Trichoderma have attracted attention because of their potential antagonistic effect in controlling other fungi (Rifai 1969; Dennis and Webster 1971a, b, c). Trichoderma is considered to be able to control Ganoderma*,* successfully (Soepena et al. 2000). It has been shown that the disease is significantly lower in a field treated by a biological control agent (*T. harzianum*) than in an untreated field (Susanto et al. 2005). It has also been shown that production of certain fungal cell wall-degrading enzymes like glucanases and chitinases increase in oil palm trees in the presence of *Trichoderma spp.* (Ferreira et al. 2007) as a defence mechanism due to the host-pathogen interaction (López 2010; Shrestha et al. 2008; Naher et al. 2011).

Filamentous fungi have chitin and β1,3-glucan in their hyphae so glucanases and chitinases have a synergistic effect on each other (Latgé 2007). The same defence reaction has previously been shown in tissues infected by *Ganoderma spp.* (Siswanto and Darmono 1998). Producing toxic secondary metabolites, however, raises serious concerns about fungal biocontrol by Trichoderma in oil palm fields (Paterson 2006b).

A wide range of endophytic bacteria from both gram positive and gram negative groups can help their host against plant pathogens through lysis or antibiotic activity. The mechanism through which these harmless bacteria induce systemic resistance (ISR) differs completely from systemic acquired resistance (SAR) in the signal transduction pathway (Kloepper and Ryu 2006). Different types of hosts from grass to woody plants take advantage of these endophytes. From the time that *Pseudomonas fluorescens* was shown to elicit ISR in cucumber against cucumber anthracnose (Wei et al. 1991) a diverse genera of bacteria mostly belonging to *Bacillus* and *Pseudomonas* have been found to elicit ISR in their hosts.

Antagonistic activity of endophytic bacteria against *G. boninense* has been studied intensively and *Burkholderiacepacia*, *Serratiamarcescens* and *Pseudomonas aeroginosa* have been introduced as candidates (Zaiton et al. 2006). In another attempt, the inhibitory effect of isolated endophytic bacteria from healthy oil palm roots on *G. boninense* was studied *in vitro*. Among 20 isolates, *P. aeroginosa* was the only identified bacteria to have a significantly strong inhibitory effect on mycelial growth in culture media (Bivi et al. 2010). In yet another approach, the effect of different isolates of *Bacillus* and Enterobacter was tested on *G. boninense* growth on oil palm seedlings (Suryanto et al. 2012).

Susceptibility differences among host populations can either be as a result of genetic resistance in the trees (Breton et al. 2006; Idris et al. 2004) or perhaps pathogenicity differences of isolates of *G. boninense* (Rees et al. 2007). It is suggested that breeding and selection of palms containing more lignin making them more resistant to the Ganoderma (Casler et al. 2002) may be another approach to the disease control and invites more investigation.

Meanwhile, the ability of trees to express glucanase and chitinase provides a potential target for genetically resistant trees. Inevitably, chitinases are used in growth and development mechanisms of tree (Santos et al. 2008). Several types of plant chitinases have been detected from different parts of various plants, and are involved in the defence mechanism against fungal pathogens (Liu et al. 2005; Ponstein et al. 1994). They have an acidic iso-electric point and accumulate in intercellular spaces (Metraux et al. 1989; Silipo et al. 2010) or a basic iso-electric point and remain in central vacuoles (Mauch and Staehelin 1989; Collinge et al. 1993). It is known that plants synthesize different isozymes of chitinase using a multigene family with diverse functionality (van Hengel et al. 2001; Zhong et al. 2002). Thus, the total chitinase activity does not necessarily relate to the host-pathogen interaction and recent studies are focussing on detection of the type of chitinase expressed as a result of fungal invasion (Yeoh et al. 2013; Naher et al. 2011) using cloning techniques and study of the gene expression. Similar studies have been implemented for glucanases (Yeoh et al. 2012).

### Resistant planting materials and screening for resistance

In the longer term, the use of resistant planting materials offers the greatest hope for the future control of BSR. Many plantations are already screening their oil palm planting materials for resistance against Ganoderma (Idris et al. 2006; Chung 2011). Methods using infested wood block inoculum have been documented by Rees and colleagues who have also shown that shading of seedlings temperature both have a dramatic effect on disease (Rees et al. 2007).

Early molecular studies have not yet isolated defence-related genes - the genetics and nature of resistance by oil palms to Ganoderma is still unknown.

Ultimately, proper management of the field remains the most important part of disease control. Huge amounts of waste created by cutting down the old or dead stands were previously burned but burning in the field can cause further problems. Nutritious debris can be a favourable culture media for infectious fungi and potentially spread the disease (Flood et al. 2000) so it is recommended removing these from the site, especially before replantation (Flood et al. 2005; Panchal and Bridge 2005).

Conversely, an oil palm trunk enriched with all the required nutrients is considered recyclable for fertilizing the field, although a small proportion of nutrients are released despite the fast degradation. The decomposition pattern shows that higher parts of the trunk have less lignin than basal segments. Using specific methods to pulverize and spread debris to accelerate degradation, waste can be managed along with Ganoderma and other pests can be reduced but unfortunately this whole process tends to be uneconomic (Paterson 2007).

As early detection is still in under debate, it is not surprising that finding a practical, successful disease control system for BSR disease appears to be equally complicated, demanding a variety of techniques and strategies.

## Conclusion

The white rot fungus, *Ganoderma boninense*, is now known as a major threat to the lucrative palm oil industry in south-east Asia. It is believed that inoculum left by the alternative host plants, the inoculum from infected trees spreading by mycelial root contact and airborne basidiospores are the three main ways this fungus spreads. Due to the shortage of data, there have been difficulties in designing an efficient rapid technique for reliable early detection but there have been some significant advances over simple observation.

Extensive work has been undertaken with PCR techniques and antibodies for the ELISA technique but the PCR technique has proven too vulnerable to contamination.

The next option, DNA-based nanosensors and DNA microarrays, have been found to be easier to operate, faster, more accurate and more economically viable than conventional PCR-based techniques. These need to be investigated further as the issue of DNA extraction and proper sampling remains a big challenge.

It is also possible that screening for secondary metabolites along with the components of fungi in the host could be an alternative technique to direct molecular methods.

In parallel, non-molecular methods such as E-nose, tomography and Hyperspectral reflective data are being investigated with some level of success but are still a long way off providing species-specific detection.

Overall, it would seem that the most promising research is molecular. DNA foot printing as well as genome sequencing is best placed to provide the data so critical for facilitating phylogenetic and systematic studies.

Until the ideal future solution of disease resistance becomes a reality, attempts to manage oil palm plantations will continue to be predominantly centred on BSR control by removing diseased material to prevent root contact from infected trees. This is done through isolation trenching, ploughing, harrowing, clearing, burning and fallowing before replanting the soil with seedlings. Sanitation remains the single most important measure at this time in the wait for Ganoderma resistant palms.

In conclusion, there is an urgent need for an intensive genome project is undertaken on *G. boninense*. This is a perfect example of a situation where the adage “prevention is better than cure” holds true. Fortunately, there has been some decrease in incidence of devastation since management of the fields has been better understood but a strong unmet demand for research into resistant oil palms and biological controls remains if the economic benefits of the palm oil industry are to be sustained in South East Asia.

## Authors’ original submitted files for images

Below are the links to the authors’ original submitted files for images. Authors’ original file for figure 1 Authors’ original file for figure 2 Authors’ original file for figure 3 Authors’ original file for figure 4

## Abbreviations

BSR: Basal stem rot
USR: Upper stem rot
EDTA: Ethylenediaminetetraacetic acid
PCR: Polymerase chain reaction
ITS: Internal transcribed spacer
RAPD: Random amplification of polymorphic DNA
RAMS: Random amplification microsatellite
RFLP: Restriction fragment length polymorphism
AFLP: Amplified fragment length polymorphism
IAC: Internal amplification control
MPOB: Malaysian palm oil board
ELISA: Enzyme-linked immunosorbent assay
FISH: Fluorescence in situ hybridization
MIP: Molecularly imprinted polymer
HPLC: High pressure liquid chromatography
LCC: Legume cover crops
ISR: Induce systemic resistance
SAR: Systemic acquired resistance.
