# Supplementary material for: Detection and control of Ganoderma boninense: strategies and perspectives
Source: Springerplus. 2013 Oct 24;2:555. doi: 10.1186/2193-1801-2-555 (PMC3824713; doi:10.1186/2193-1801-2-555)
Supplement: Supplementary file 1 — Authors’ original file for figure 1 [file 40064_2013_610_MOESM1_ESM.pdf]

```
                  1                                                                                                                                   135
BD082757      (1)
BD082758      (1)
BD082759      (1)                                          A                                                           G                      ----
Consensus     (1) AAGGATCATTATCGAGTTTTGACTGGGTTGTAGCTGGCCTTCCGAGGCATCGTGCACGCCCTGCTCATCCACTCTACACCTGTGCACTTACTGTGGGTTATAGATCGTGTGGAGCGAGCTCGTTCGTTTGACGAG
                  136                                                                                                                                 270
BD082757    (136)
BD082758    (136)                                                                                                                      C
BD082759    (132)   T
Consensus   (136) TTCGCGAAGCGCGTCTGTGCCTGCGTTTTATCACAAACACTATAAAGTATTAGAATGTGTATTGCGATGTAACGCATCTATATACAACTTTCAGCAACGGATCTCTTGGCTCTCGCATCGATGAAGAACGCAGCG
                  271                                                                                                                                 405
BD082757    (271)                 T
BD082758    (271)
BD082759    (267)
Consensus   (271) AAATGCGATAAGTAATGTGAATTGCAGAATTCAGTGAATCATCGAATCTTTGAACGCACCTTGCGCTCCTTGGTATTCCGAGGAGCATGCCTGTTTGAGTGTCATGAAATCTTCAACCTACAATCTCTTTGCGGT
                  406                                                                                                                                 540
BD082757    (406)
BD082758    (406)
BD082759    (402)
Consensus   (406) TTTTGTAGGCTTGGACTTGGAGGCTTGTCGGTCTTTTATTGATCGGCTCCTCTCAAATGCATTAGCTTGGTTCCTTTGCGAATCGGCTGTCGGTGTGATAATGTCTACGCCGCGACCGTGACGCGTTTGGCGAGC
                  541                                                                      616
BD082757    (541)
BD082758    (541)                            G
BD082759    (537)
Consensus   (541) TTCTAACCGTCCCGTTATTGGGACAACTCTTATGACCTCTGACCTCAAATCAGGTAGGACTACCCGCTGAACTTAA
```
