# Supplementary figures and images for: Detection and control of Ganoderma boninense: strategies and perspectives
Source: Springerplus. 2013 Oct 24;2:555. doi: 10.1186/2193-1801-2-555 (PMC3824713; doi:10.1186/2193-1801-2-555)

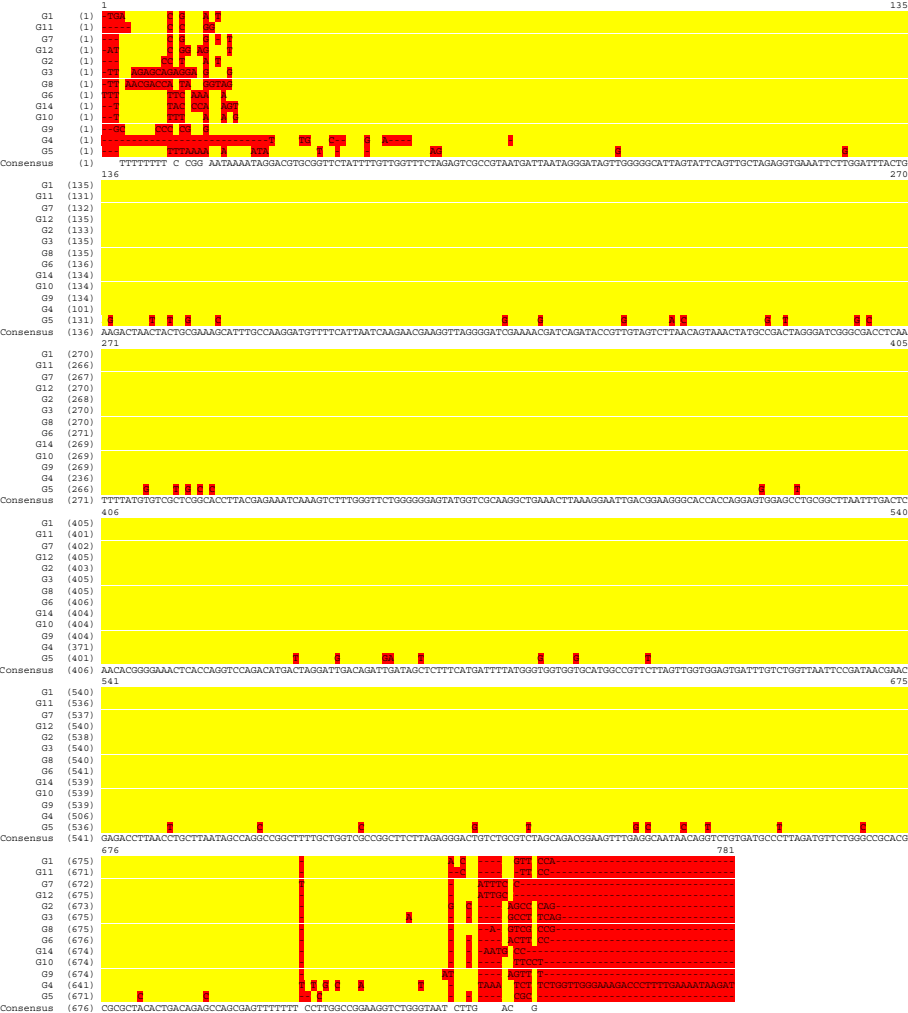

Supplement: Supplementary file 2 — Authors’ original file for figure 2 [file 40064_2013_610_MOESM2_ESM.pdf]

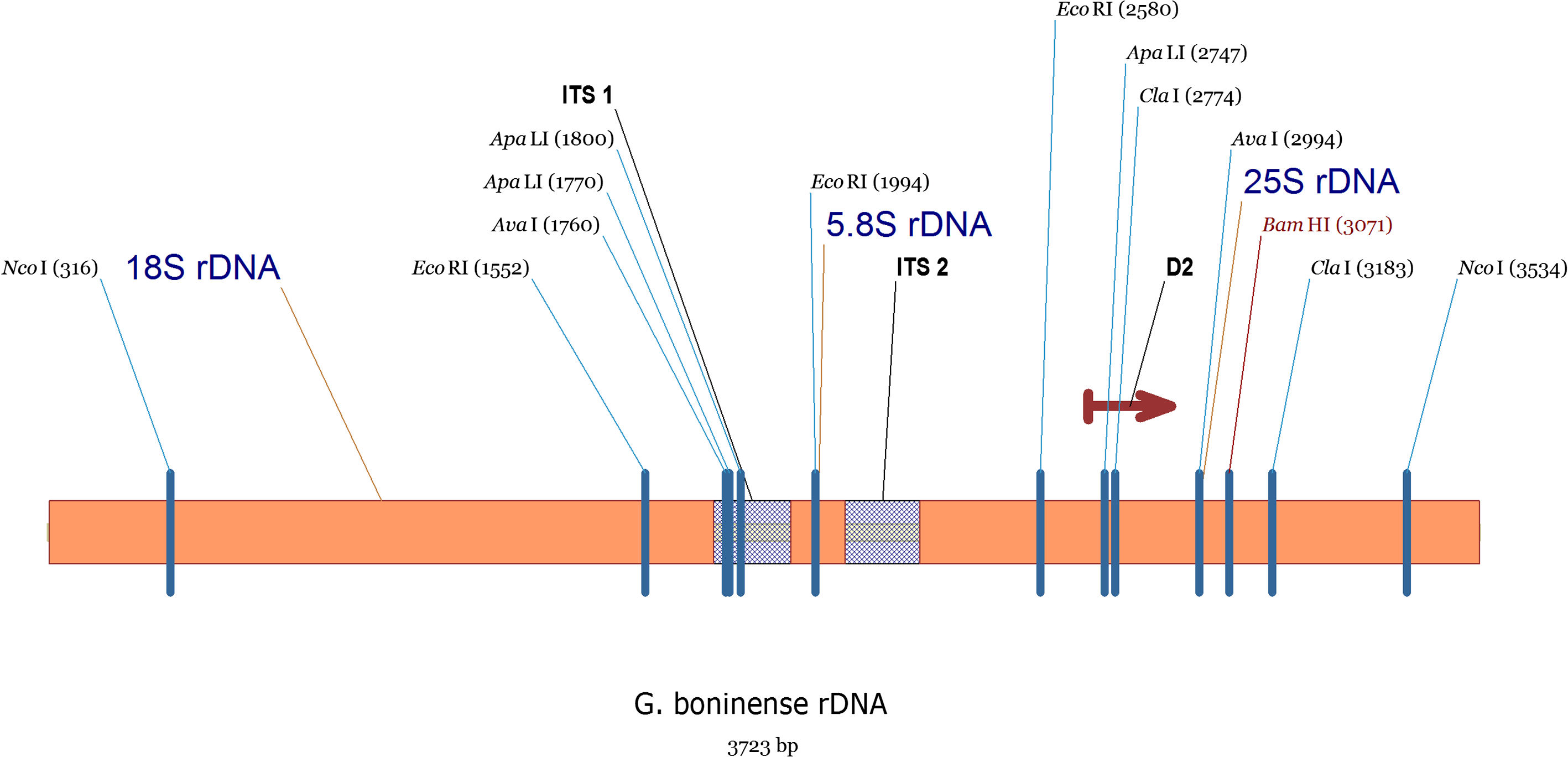

Supplement: Supplementary file 4 — Authors’ original file for figure 4 [file 40064_2013_610_MOESM4_ESM.tif]
