# Supplementary material for: Detection and control of Ganoderma boninense: strategies and perspectives
Source: Springerplus. 2013 Oct 24;2:555. doi: 10.1186/2193-1801-2-555 (PMC3824713; doi:10.1186/2193-1801-2-555)
Supplement: Supplementary file 3 — Authors’ original file for figure 3 [file 40064_2013_610_MOESM3_ESM.pdf]

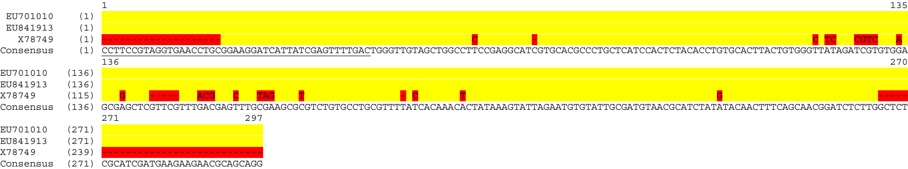
1
135
EU701010 (1)
EU841913 (1)
X78749 (1)
------------------- C - C TC CGTC A
Consensus (1) CCTTCCGTAGGTGAACCTGCGGAAGGATCATTATCGAGTTTTGACTGGGTTGTAGCTGGCCTTCCGAGGCATCGTGCACGCCCTGCTCATCCACTCTACACCTGTGCACTTACTGTGGGTTATAGATCGTGTGGA
136
270
EU701010 (136)
EU841913 (136)
X78749 (115)
G ----- ACG C TAG T - C T G -----
Consensus (136) GCGAGCTCGTTCGTTTGACGAGTTTGCGAAGCGCGTCTGTGCCTGCGTTTTATCACAAACACTATAAAGTATTAGAATGTGTATTGCGATGTAACGCATCTATATACAACTTTCAGCAACGGATCTCTTGGCTCT
271
297
EU701010 (271)
EU841913 (271)
X78749 (239)

Consensus (271) CGCATCGATGAAGAAGAACGCAGCAGG
